# Flexure Performance of Ferrocement Panels Using SBR Latex and Polypropylene Fibers with PVC and Iron Welded Meshes

**DOI:** 10.3390/polym15102304

**Published:** 2023-05-14

**Authors:** Hisham Jahangir Qureshi, Nauman Khurram, Usman Akmal, Md Arifuzzaman, Muhammad Qamar Habib, Abdulrahman Fahad Al Fuhaid

**Affiliations:** 1 Department of Civil and Environmental Engineering, College of Engineering, King Faisal University, Al-Ahsa 31982, Saudi Arabia; marifuzzaman@kfu.edu.sa (M.A.); aalfuhaid@kfu.edu.sa (A.F.A.F.); 2 Department of Civil Engineering, University of Engineering and Technology Lahore, G.T. Road Baghbanpura, Lahore 39161, Pakistan; nauman@uet.edu.pk (N.K.); usman.akmal@uet.edu.pk (U.A.); 2018civ73@student.uet.edu.pk (M.Q.H.)

**Keywords:** ferrocement, plastic mesh, polypropylene fibers, styrene–butadiene latex, sustainable construction material, flexure strength, low-cost construction, light weight construction

## Abstract

Ferrocement panels are thin-section panels that are widely used in lightweight construction. Due to lesser flexural stiffness, they are susceptible to surface cracking. Water may penetrate through these cracks and may cause corrosion of conventional thin steel wire mesh. This corrosion is one of the major factors which affect the load-carrying and durability of ferrocement panels. There is a need to improve the mechanical performance of ferrocement panels either through using some non-corrodible reinforcing mesh or through improving the cracking behavior of the mortar mix. In the present experimental work, PVC plastic wire mesh is employed to address this problem. SBR latex and polypropylene (PP) fibers are also utilized as admixtures to control the micro-cracking and improve the energy absorption capacity. The main idea is to improve the structural performance of ferrocement panels that may be utilized in lightweight, low-cost house construction and sustainable construction. The ultimate flexure strength of ferrocement panels employing PVC plastic wire mesh, welded iron mesh, SBR latex, and PP fibers is the subject of the research. Test variables are the type of mesh layer, the dosage of PP fiber, and SBR latex. Experimental tests are conducted on 16 simply supported panels of size 1000 × 450 mm and subjected to four-point bending test. Results indicate that the addition of latex and PP fibers only controls the initial stiffness and does not have any significant effect on ultimate load. Due to the increased bonding between cement paste and fine aggregates, the addition of SBR latex improves the flexural strength by 12.59% and 11.01% for iron mesh (SI) and PVC plastic mesh (SP), respectively. The results also indicate an improvement in the flexure toughness of specimens with PVC mesh as compared to specimens with iron welded mesh; however, a smaller peak load is observed (i.e., 12.21% for control specimens) compared with the specimen with welded iron mesh. The failure patterns of the specimens with PVC plastic mesh exhibit a smeared cracking pattern that shows that they are more ductile compared to samples with iron mesh.

## 1. Introduction

Ferrocement is a member of a structural concrete family, which uses a combination of thin reinforcing bars and layers of mesh that are closely placed and embedded in mortar. Steel mesh is the most commonly used reinforcement, but metallic meshes may be combined with other natural or synthetic materials. The mortar is generally a mixture of ordinary Portland cement and sand or any other fine aggregates with a particle size of less than 2.36 mm (passing through a no. 8 sieve) [1]. The availability of materials and ease of fabrication with minimum technical skill has made ferrocement quite popular around the world, and this technique is extensively being used in water and food storage facilities, housing units, botas, shell roofing system, and prefabricated building members. The specific surface area of reinforcement in ferrocement is significantly higher than conventional reinforced concrete cement (RCC), which increases the ductility and reduces the thickness of the panel, thus reducing the total weight [2]. Through utilizing proper curing techniques, ferrocement slabs can become sturdy, impermeable, and resistant to stress, abrasion, freezing, thawing, and breaking from shrinkage and self-weight. Further, the lesser width of the meshing reinforcement increases the performance of the ferrocement panel via controlling crack width and intensity [3,4,5]. Less steel reinforcement and cement content as compared to standard reinforced concrete masonry construction makes ferrocement a more environmentally friendly and sustainable material in terms of construction cost, time, and CO_2_ generation per unit of floor area [6].

In 1852, Lambot fabricated rowing boat steel mesh and enriched cement mortar and named that compound as Feri-cement. The idea of ferrocement was further revived by Nervi [7], and it was investigated that stacking the wire meshes in mortar makes the mechanical characterization of ferrocement virtually homogenous, which also increases its impact resistance. Rapid economic growth, urban settlement development, and expansion are increasing the demand for new housing units in the construction industry. Due to high demand for housing and rising construction costs, housing has become an expensive option, even on a global scale [8]. High mechanical characterization with lighter weight makes ferrocement an economical alternative for housing roofing systems [9]. Moreover, ferrocement may also be used for the retrofitting and strengthening of existing structures [10].

Natural or man-made disasters, i.e., floods and earthquakes, demand robust and cheap housing construction for affected people, and in such cases precast ferrocement panels may be an ideal alternative solution. Due to smaller thickness as compared to conventional RCC members, ferrocement panels have lesser flexural stiffness and they are more susceptible to cracking at the service stage. Moreover, the low tensile strength of mortar and shrinkage at early ages may cause micro-cracking in ferrocement panels [11,12]. These micro-cracks may propagate over time and may cause durability problems if moisture or rainwater infiltrate inside the panel through these cracks. This moisture causes corrosion of the thin reinforcing mesh and leads to strength reduction [13]. This issue can be addressed either through controlling the surface cracking via incorporating fibers and energy absorption materials into the mortar or using some non-corrodible reinforcing mesh. Further, the deterioration of the durability and capacity of ferrocement panels due to chemical attachment and corrosion may also be resolved through using high-molar geopolymer-based mortar [14]. Many researchers have used non-corrodible reinforcing mesh, i.e., polypropylene and PVC-coated meshes, and compared the results with panels with GI-coated steel weld mesh, and their results indicated that ferrocement panels with these meshes could be used as an alternative solution to reinforcement corrosion [13,15,16]. In this regard, Rameshkumar et al. [17] used polypropylene warp knitted fabric in ferrocement panels, replacing the conventional iron welded mesh. Flexural strength and energy absorption were the main parameters investigated in the study. The experimental results indicated that polypropylene warp knitted fabrics improve the first crack load and peak load by 200% and 120%, respectively, as compared with the control specimens and, thus, can be used as non-corrodible reinforcement in ferrocement panels.

Mousavi et al. [18] observed the performance of steel-fiber-reinforced ferrocement panels through replacing cement with silica fume, using one, two, and three layers of galvanized wire meshes. High-strength hooked-end steel fibers having a diameter of 0.55 mm and length of 25 mm were used in the study. The specimens were tested against central point flexural loading. The experimental results exhibited that the flexural strength and deflection were increased, and lateral face crack width decreased as the number of reinforced meshing layers increased. Moreover, a 15% dosage of silica fume provided the optimal mechanical properties. Yaswanth et al. [19] investigated the effect of carbon fibers on the bending strength of ferrocement panels. Four slab panels of size 700 mm × 600 mm × 30 mm with and without carbon fibers were prepared in the study. A 40 MPa mortar with 1% carbon fiber and welded square mesh were incorporated in casting the panels. The panels with 1% carbon fiber showed better crack control during flexure testing. The study also reported that adding polypropene (PP) fibers in an amount of 0.4% can improve the flexural strength of ferrocement panels by up to 80%.

Many studies have also indicated that steel fibers in ferrocement panels can be used to improve the first crack load, ultimate capacity, and ductility [20,21]. Jaafar [22] investigated the effect of a slurry infiltrated fiber (SIFCON) matrix on the energy absorption, fracture patterns, and ultimate load of fourteen ferrocement panels of size 500 mm × 120 mm × 20 mm. Four different percentages of steel fibers (i.e., 2.5%, 5%, 7.5% and 10%) with a welded square mesh of 1.0 mm diameter, 12.5 mm spacing, and 405 MPa yield strength were used in the ferrocement panels. Mortar matrixes also consisted of 10% silica fume by weight of cement and hook-ended steel fiber having a diameter of 1.0 mm and a length of 60 mm. The experimental results showed an appreciable increase in energy absorption and peak load. Some researchers have also utilized admixture and high-performance chemicals to improve energy dissipation and initial cracking load. In this regard, SBR latex is one of the most widely utilized polymers in concrete, as it boosts the material’s flexural, tensile, and bond strengths when compared to conventional concrete [23,24]. Isikidag [25] utilized expanded perlite-based mortar in twelve ferrocement panels of 900 mm × 300 mm × 50 mm size with square woven mesh and hexagonal woven meshes, and tested them against four-point loading. The results depicted that the duration of the initial crack and final crack decreases as the number of layers and cement content increase.

The previous studies conducted on ferrocement and cited references indicate that the flexure stiffness, micro-cracking, and energy absorption characteristics of ferrocement panels can be improved through utilizing fibers and/or using admixture of materials such as SBR latex. Moreover, durability and strength reduction may be controlled via incorporating some non-corrodible reinforcing mesh. In the present study, the effect of SBR latex and PP fibers on energy absorption, load carrying capacity, and failure behavior of ferrocement panels using two different types of reinforcing mesh, i.e., welded iron steel and PVC plastic materials, were examined. The four-point bending test was used to assess the application of these panels to be used as partition/boundary walls and a non-load-bearing and lightweight roofing system. The present study aims to develop ferrocement panels that are more durable, free from reinforcement corrosion, and have more energy absorption characteristics for low-cost housing systems and the development of sustainable infrastructure.

## 2. Materials and Methods

### 2.1. Marix Componenets

**Figure 1:**
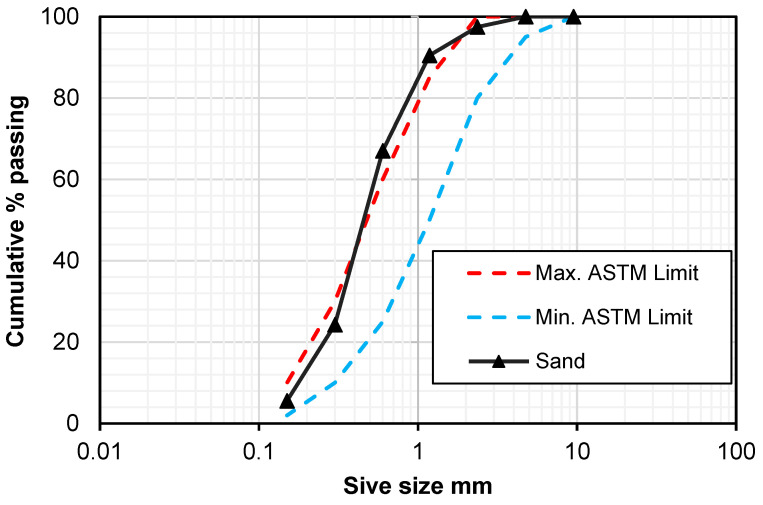
Gradation curve of sand.

ACI-549R-18 [1] recommends the utilization of sand passing through a no. 8 sieve (2.36 mm); cementitious material, usually ordinary Portland cement (OPC); and water to prepare the matrix for ferrocement. An OPC conforming to type 1 of ASTM C-150 [26] and having a specific gravity of 3.12 was used for the preparation of mortar mix. A locally available pit sand satisfying the limiting values of ASTM C-136 [27] was utilized as a fine aggregate. Table 1 shows the chemical composition of cement and sand. More than 97.5% of the sand used in the present experimental study was passed through the no. 8 sieve. The particles size distribution and physical properties of the sand are presented in Figure 1 and Table 2, respectively.

### 2.2. Reinforcement

**Figure 2:**
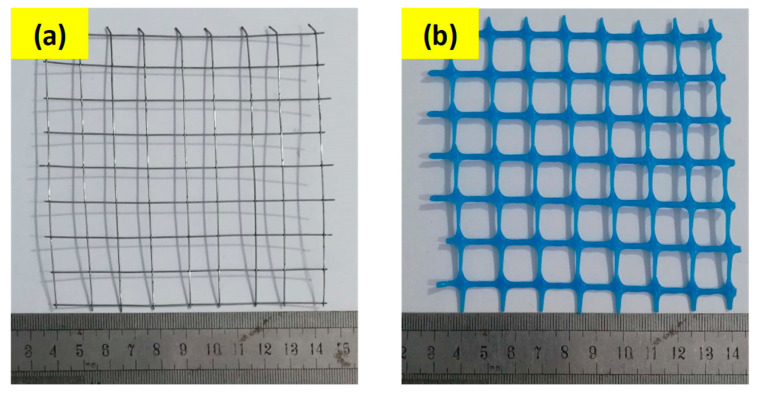
Reinforcing mesh used in ferrocement panels: (**a**) PVC plastic mesh and (**b**) iron welded mesh.

ACI committee 549 [31] highlighted that a fine rectangular reinforcing mesh placed in normal orientation (0° and 90° with respect to panel length) is more effective compared to any other mesh shape or mesh orientation. Due to this reason, two different types of wire mesh with square openings, i.e., welded iron and PVC plastic meshes, as shown in Figure 2, were used as the reinforcement in ferrocement panels. Mechanical properties of both meshes are given in Table 3. To maintain the spacing and keep the reinforcing mesh in a stretched position, a plane or ribbed bar made of steel or any other natural material of 6–10 mm diameter can be used as skeletal reinforcement in either side of the ferrocement panel [1,32]. Two steel rebars of 6 mm diameter were also used as skeleton steel in the longitudinal direction only. The ASTM A615M [33] was used to compute the yield and ultimate strength as 427 MPa and 571 MPa, respectively.

### 2.3. Polypropylene Fibers

Polypropylene ferrocement panels are thin in nature and have a minimal reinforcement cover thickness (i.e., 3–5 mm). Due to this reason, fibers in conjunction with reinforcing mesh can be used to improve mechanical properties of ferrocement panels, especially in the post-cracking phase. Polypropylene (PP) fibers are rust-free and are compatible with all types of chemical admixtures. Due to the chemical inertness, PP fibers are safe to handle and do not pose any harmful effect on concrete. Owing to their hydrophobic nature, PP fibers do not get moist, which removes the minimum cover requirement in concrete and mortar. In the current experimental study, a constant dosage of PP fibers equal to 0.5% by weight of cement was used as suggested by reference studies [34,35]. Table 4 shows the technical details of PP fibers as provided by the manufacturer.

### 2.4. SBR Latex

**Figure 3:**
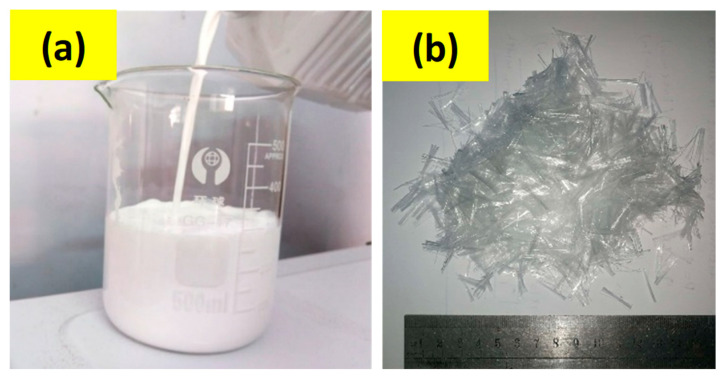
Admixtures used in mortar mix: (**a**) SBR latex and (**b**) PP fibers.

SBR (styrene–butadiene rubber) latex is a synthetic rubber emulsion, which is used to improve the strength of cement mortars and concrete by providing superior adhesion and water resistance. It is a powerful binding agent with significant crack-bridging characteristics. In the present study, a fixed dosage of SBR latex, 5% of cement weight conforming to ASTM C1042 [36] as recommended by the manufacturer, was utilized. Figure 3 shows the SBR latex and PP fibers used in the present study, whereas the technical characteristics of SBR latex are provided in Table 5.

### 2.5. Mix Proportion

In order to obtain mortar strength within the range of 35 to 50 MPa for ferrocement panels, the mix design as shown in Equation (1) was used in all mixes, and that also is in accordance with limits defined by ACI-549 [1] for mortar mixes (i.e., cement:sand = 1.4–2.5 and water:cement = 0.35–0.6). This mix design has also been used in similar studies [13,16]. Along with the normal mix (referred to as control mix), there different mixes were also prepared. In two mixes, 0.5% PP fibers and 5% of SBR latex by weight of cement were added separately, while in the third mix, both PP fibers and SBR latex were included to investigate their combined effect. The mixing was carried out in a high-speed vertical-rotary type of concrete mixer and clean tap water, free from any organic impurities, was used in mixing. At first, cement and sand were placed in the mixer, and dry mixing was carried out for 2–3 min. The PP fibers were sprinkled steadily during dry mixing to assure uniform distribution. In the next step, the water was added gradually, and mixing was continued for another 1–2 min to obtain a homogeneous mortar mix. For mixes with latex, the SBR latex was replaced with an equal amount of water to maintain a constant water cement ratio and then mixed with water to be included during wet mixing. After mixing, three cubes of 50 × 50 × 50 mm from each mix were cast to determine their compressive strength.

(1) C:S:W=1:2:0.5

### 2.6. Type of Specimens and Casting

Four different types of panels, i.e., the control specimen (C), specimens with PP fibers (F), specimens with SBR latex (S) and specimens with both fibers and SBR latex (SF) were prepared for each type of mesh, i.e., plastic mesh (P) and iron mesh (I). In order to obtain more reliable results, two panels of each type were cast. The detail and nomenclature of each type of specimen are mentioned in Table 6. As mentioned earlier, dosage of PP fiber and SBR latex was decided as 0.5% and 5% by weight of cement. Citing the reference studies [13], the panel size was decided as 1000 mm × 450 mm with a total thickness equal to 40 mm. A minimum clear cover of 5 mm was decided to protect the welded iron mesh from corrosion [37].

### 2.7. Preparation of Formwork

**Figure 4:**
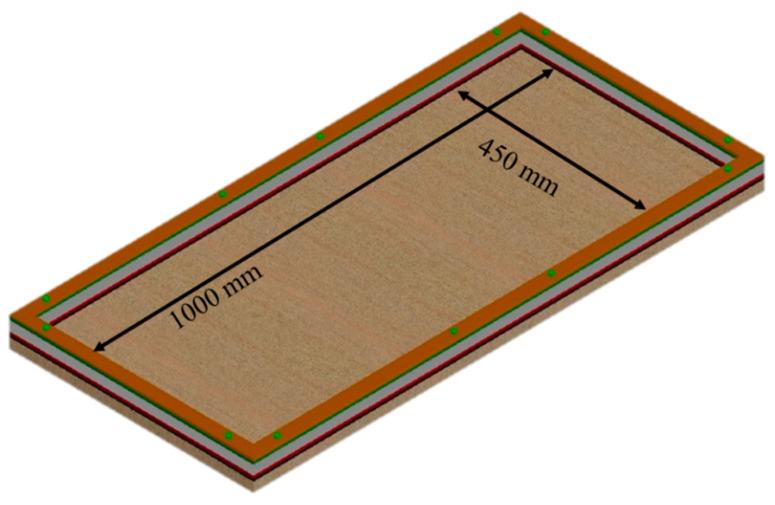
Schematic diagram of formwork.

**Figure 5:**
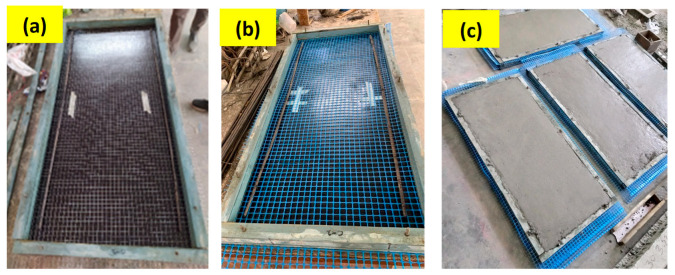
Casting process of ferrocement panel: (**a**) formwork with steel mesh, (**b**) formwork with plastic mesh, and (**c**) panels after complete pouring of mortar.

For casting the ferrocement panels, a special formwork was fabricated; the internal dimensions of the formwork are shown in Figure 4. The thickness of the formwork was comprised of three plies. In order to maintain the clear cover, the thickness of the outer two plies was kept equal to 5 mm, whereas the thickness of the middle ply was kept equal to 30 mm to make the total thickness of the panel 40 mm. The casting of mortar into the panel was performed on a vibrating table, and before casting, the formworks were cleaned and oiled. The casting was performed stepwise. First, a ply of 5 mm was placed on the formwork plate, and then the first meshing layer was placed. The reinforcing mesh size was kept around 75 mm more on each side of the formwork for easy stretching and to avoid sagging during the pouring of mortar. In the next step, a second ply of 30 mm was placed, and two longitudinal steel bars of 6 mm were provided as skeletal reinforcement at mid-height in the longitudinal direction only. The mortar was poured gradually in layers until it reached a depth of 35 mm and was then vibrated for a few seconds to attain proper compaction by removing air bubbles. Afterward, the second layer of reinforcing mesh was placed in a stretching state and the remaining ply of 5 mm was placed over the formwork. Lastly, the remaining thickness was filled with mortar and compacted to have a smooth surface. Figure 5 shows formwork panels with iron mesh and plastic mesh before and after casting. The formwork was opened after 24 h, extra mesh was ground using a cutter, edges were plastered, and panels were cured for 28 days by placing them in moistened jute bags.

### 2.8. Testing Procedure

**Figure 6:**
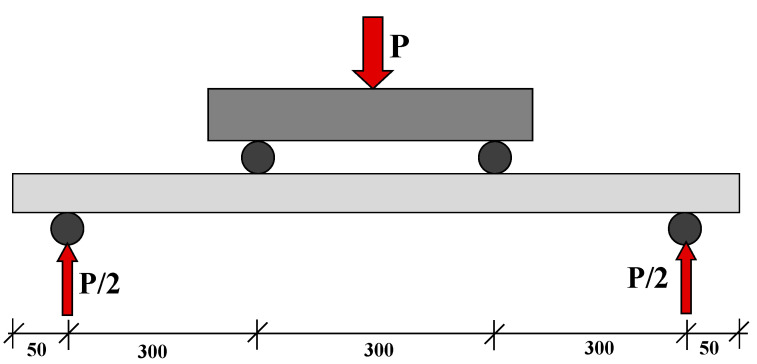
Schematic diagram of testing setup (dimensions are in mm).

**Figure 7:**
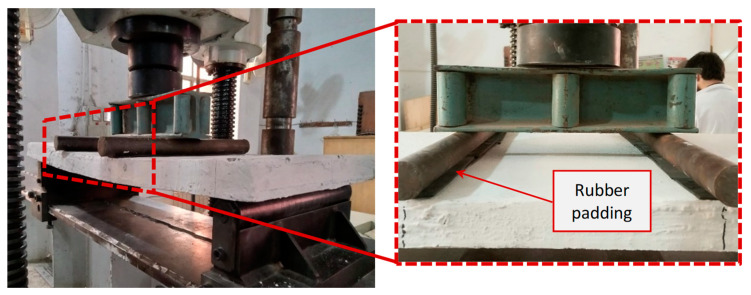
Experimental setup for flexure test.

The ferrocement panels were tested for flexure using a four-point bending test. The total span and loading positions are illustrated in Figure 6. The bending test was performed on a fully calibrated and computerized universal testing machine (UTM). The load (P) was applied using a displacement control procedure with a loading rate of 1 mm/s. The testing data were recorded in terms of machine stroke (mm) versus load in kN through the built-in data acquisition system in the UTM. Rubber padding was used under the roller pins to assure a uniform application of the load on the panel surface. The complete experimental step is shown in Figure 7.

## 3. Results

### 3.1. Compressive Strength

**Figure 8:**
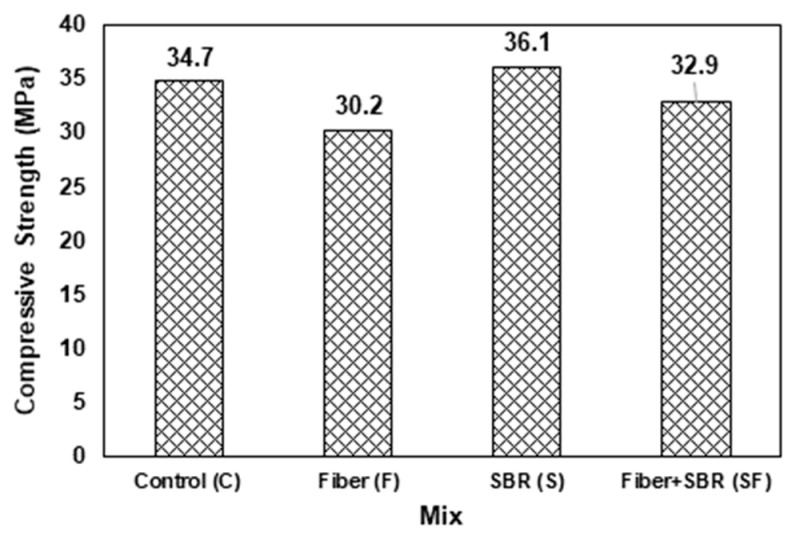
Compressive strength of mortar mixes.

The compression test was performed on three cube specimens of each mix type, and their average results are presented in Figure 8. The control specimens (C) indicated a compressive strength of 34.7 MPa. The inclusion of 0.5% PP fibers (F) reduced the strength to 30.3 MPa, which is mainly attributed to the reduction in density and increment of the interfacial transition zone in the mortar mix [38]. The high binding and crack-bridging characteristics of SBR latex contribute to the compressive strength of the concrete and mortar mix [23,39], and due to this reason, the compressive strength of mix (S) with SBR latex improved to 36.1 MPa. Since the addition of PP fibers has an adverse effect on compressive strength, a hybrid mix (SF) was also prepared via adding 5% SBR latex along with 0.5% PP fibers by weight of cement. The results for the hybrid mix (SF) indicated an improvement of 8.94% (32.9 MPa vs. 30.2) in its compressive strength as compared to the mix with PP fibers (F) only; however, strength was still around 5.18% less than the control mix (c).

### 3.2. Load Displacement Curves for Panels with Iron Mesh

**Figure 9:**
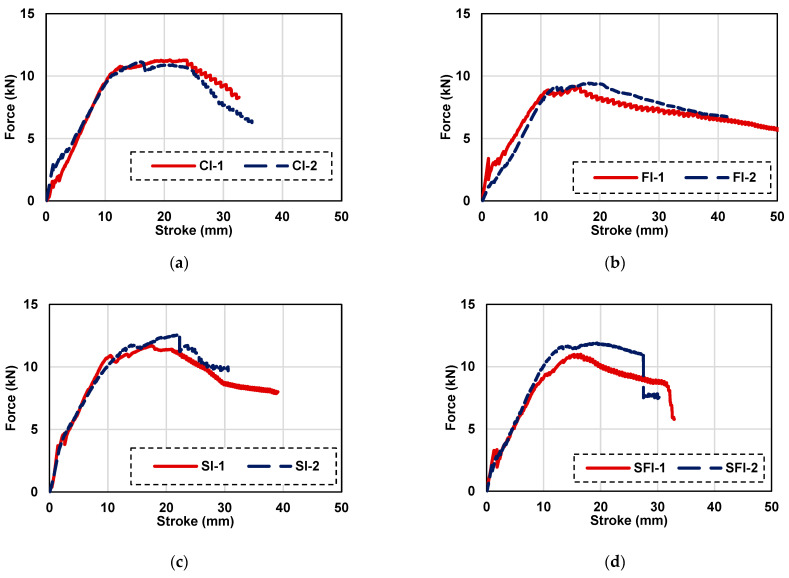
Load displacement curves of ferrocement panels with iron mesh: (**a**) control, (**b**) with PP fibers, (**c**) with SBR latex, and (**d**) with PP fibers and SBR latex.

**Figure 10:**
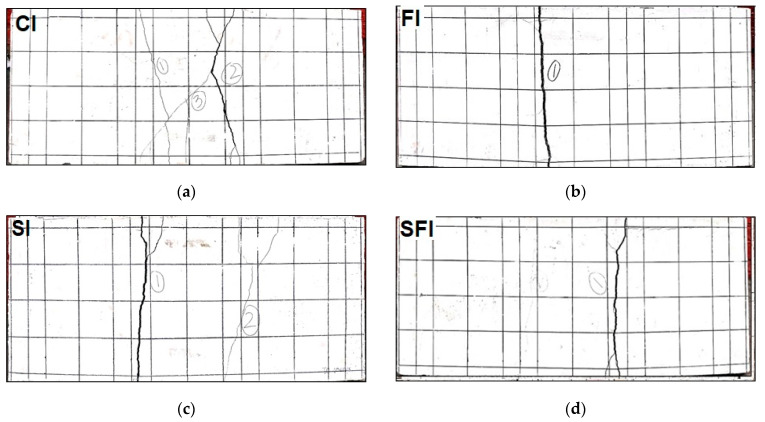
Fracture pattern of ferrocement panels with iron mesh: (**a**) control, (**b**) with PP fibers, (**c**) with SBR latex, and (**d**) with PP fibers and SBR latex.

The load-displacement responses of four-point bending tests for ferrocement panels reinforced with iron mesh are presented in Figure 9. Test results of steel mesh ferrocement panels for the control panel (CI), the panel with PP fibers (FI), the panel with SBR latex (SI), and the panel with both fibers and SBR latex (SFI) are given in Figure 9a–d, respectively. For flexure, displacement-controlled loading was applied at 1 mm/min. Results are presented for two specimens prepared using iron mesh. For all mixes, in steel mesh ferrocement specimens, the initial linear part of the curve is up to the point when cracking in the mortar occurs at the tension face, as shown in Figure 10. After cracking of the outer surface, additional strength is contributed by the steel mesh and load carrying capacity is increased to peak load. The observed fracture phases are the same as those reported by various prior research studies [13,16]. The test was stopped when the post-peak load dropped to around 80 percent of the maximum load. The peak load of each specimen for each mix is summarized in Table 7. Peak average load and its percentage difference (increase/decrease) with respect to control is also presented in Table 7. The average peak flexural load dropped by about 17 percent with reference to the control mix when polypropylene fibers were added to the mortar mix. One of the reasons for this decrease in strength is related to the interfacial bond between the fibers and the cement matrix. Polypropylene fibers tend to have a smooth surface, which can make it difficult for them to bond well with the cement matrix. This weakens the interfacial bond, making the composite material less strong and less durable. Similar results regarding a drop in the flexural strength of concrete and mortars have been reported in past studies [38,40]. On the other hand, the average peak flexural load of ferrocement panels with steel mesh was increased by about 8.2 percent with respect to the control specimen when SBR latex was added in the mortar mix. The addition of SBR latex to the cement matrix can improve the bonding between the cement and the steel reinforcement, resulting in increased strength and ductility of the ferrocement panels. The polymer particles of SBR latex can fill the pores in the cement matrix, leading to improved density and reduced permeability of the composite. This can reduce the risk of cracking and increase the durability of the ferrocement panels.

Moreover, SBR latex can also enhance the flexural strength of the ferrocement panels by providing a more uniform distribution of stresses throughout the panel. The polymer particles in the SBR latex can act as bridges between the cement matrix and the steel reinforcement, resulting in a more efficient load transfer and a more balanced stress distribution [24]. The average peak flexural load of the specimens including the SBR latex and polypropylene fibers is 11.45 kN for ferrocement panels with steel mesh. This value is less than the average peak flexural load of 12.14 kN for the steel mesh ferrocement panels with the addition of SBR latex alone. The reasons are same as explained above for the difference between control and steel mesh ferrocement panels with polypropylene fibers only.

### 3.3. Load Displacement Curves for Panels with PVC Plastic Mesh

**Figure 11:**
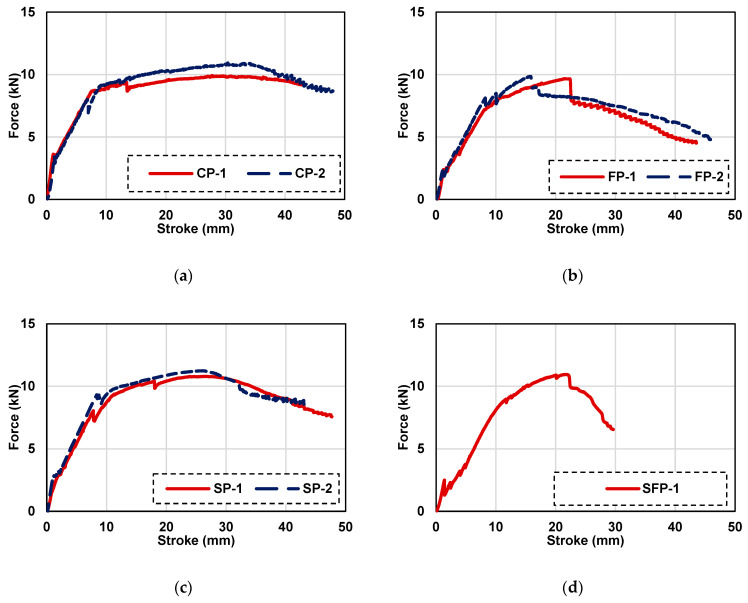
Load displacement curves of ferrocement panels with iron mesh: (**a**) control, (**b**) with PP fibers, (**c**) with SBR latex, and (**d**) with PP fibers and SBR latex.

**Figure 12:**
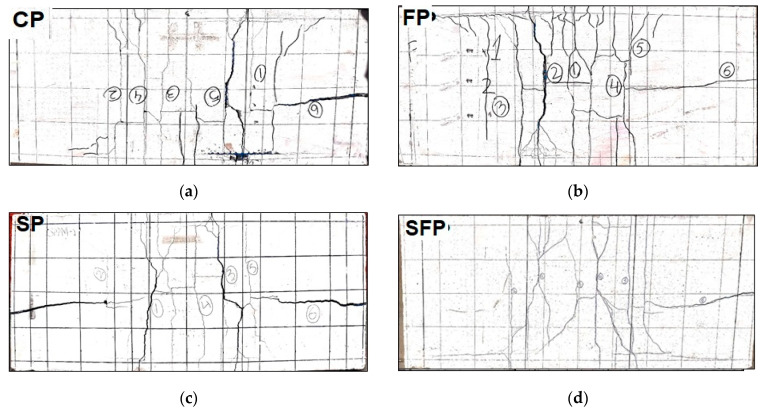
Fracture pattern of ferrocement panels with PVC plastic mesh: (**a**) control, (**b**) with PP fibers, (**c**) with SBR latex, and (**d**) with PP fibers and SBR latex.

The load displacement curves of ferrocement panels reinforced with plastic mesh are presented in Figure 11. Test results of plastic mesh ferrocement panels for control panels (CP), panels with polypropylene fibers (FP), panels with SBR latex (SP), and panels with both fibers and latex (SFP) are given in Figure 11a–d, respectively. Displacement-controlled loading was applied at 1 mm/minute. Results are presented for two specimens prepared using plastic mesh. For the mix with SBR latex and polypropylene fibers with plastic mesh, only one specimen’s results are given. The data of the second specimen could not be recorded due to a power outage during the test. For all mixes, transverse cracks initially appeared in the central zone of the ferrocement specimen slab, as shown in Figure 12, which causes the loss of stiffness due to the fracture of the mortar. After cracking of the outer surface, additional strength is contributed by the plastic mesh, and load carrying capacity is increased to peak load. After first cracking, due to the large ductility of plastic mesh, the stress is redistributed in a low-stress region and results in smeared cracking, as shown in Figure 12. This shows the more ductile behavior and higher energy dissipation characteristics of plastic mesh. It is also evident when the load displacement curves of plastic mesh panels are compared with steel mesh panels, having more post peak response. It can also be observed from the figure that for each mix, both specimens behave almost identically. The peak load of each specimen for each mix is summarized in Table 7. Peak average load and its percentage difference (increase/decrease) with respect to control are also presented in Table 7.

The average peak flexural load dropped by about 6.4 percent with reference to the control mix when polypropylene fibers were added to the mortar mix. The reasons for the drop in peak load are the same as explained in the previous section. On the other hand, the average peak flexural load of ferrocement panels with SBR latex added in the mortar mix was increased by about 11 percent with respect to the control specimen. The phenomenon of an increase in average peak load is also explained in the previous section. The average peak flexural load of the specimens including the SBR latex and polypropylene fibers is 10.94 kN for ferrocement panels with plastic mesh. This value is less than the average peak flexural load of 11.01 kN for the ferrocement panels with the addition of SBR latex alone. The reasons are the same as explained above for the difference between the control panels and plastic mesh ferrocement panels with polypropylene fibers only.

### 3.4. Flexural Toughness of Ferrocement Panels

**Figure 13:**
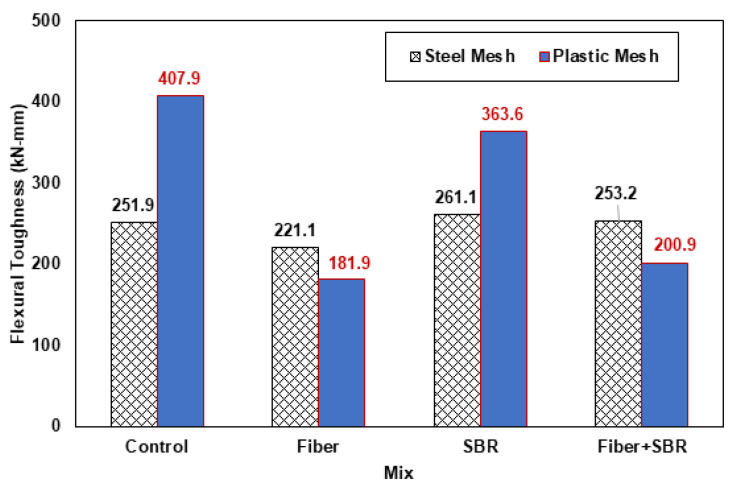
Flexural toughness results of ferrocement panels.

The flexural toughness was calculated for all specimens of ferrocement panels with steel and plastic mesh, corresponding to the area under the load displacement curves. For this purpose, curves were used from origin to 0.8 times the peak load after maximum load. The results are summarized in Table 8 with average flexural toughness and with percent difference with respect to control specimens. Average flexural toughness results are compared for both steel and plastic mesh ferrocement panels in Figure 13. Average flexural toughness is increased by about 62% for the control plastic mesh panels (407.9 kN-mm) than the control steel mesh panels (251.9 kN-mm). Additionally, average flexural toughness of plastic mesh panels is about 39.2% more with SBR latex (363.6 kN-mm) than corresponding steel mesh panels (261.1 kN-mm), whereas in other cases, it was reduced for plastic mesh panels. One thing to note is that peak load was less in cases of plastic mesh panels than steel mesh panels. However, the maximum displacement values were higher in all plastic mesh ferrocement panels than in those with steel mesh. To improve the flexural toughness of plastic mesh ferrocement panels, skeleton steel bars may also be used.

## 4. Discussion of Results

The flexural compressive strength of mixes containing polypropylene fibers was about 13% less than that of the control mix. Additionally, for mortar with SBR latex, the compressive strength increased by about 4% compared to that of control mortar. Compressive strength decreased by about 9% when PP fibers were added in addition to SBR latex compared to the mortar with only SBR latex. PP fibers are widely used in cementitious materials as reinforcement to improve flexural toughness, ductility, and impact resistance [41]. However, its effect on compressive strength is not yet clear. Past research [42,43] showed that there could be no or little chemical adhesion between the inert PP fibers and the surrounding matrix. Furthermore, the smooth surface of PP fibers enhances this effect. Moreover, a wall effect is present when fibers are added to cementitious mortars due to the formation of a water film at the interface of fiber and matrix. This process further reduces the bond between PP fibers and the surrounding matrix paste. The findings of the current study are in line with the results of the above-mentioned research works. On the other hand, positive effects were found for compressive strength with the addition of SBR latex. SBR latex improves the properties of the cement paste in the mortar and enhances the paste–aggregate bonding strength that resulted in the final properties of the SBR-modified concrete [44]. The results shown in similar studies [45,46] showed the mixed behavior of increasing and decreasing compressive strength with the increase in SBR latex content.

The peak load of ferrocement panels in flexural tests showed the same trend as the compressive strength of mortars when mixed with PP fibers and SBR latex. The peak flexural load of the mix containing polypropylene fibers was about 17% and 6% less than that of the control mix for panels reinforced with steel and PVC mesh, respectively. Additionally, for mortar with SBR latex, the peak load increased by about 8% and 5.5% compared to that of control mortar for panels with steel and PVC mesh, respectively. Furthermore, the peak loads were decreased by about 5.6% and 0.6% when PP fibers were added in addition to SBR latex compared to panels with only SBR latex for steel and PVC mesh, respectively, as given in Table 7. The reason for the decrease in peak flexural load after the addition of PP fibers is a lack of a bond between the fibers and the matrix as a result of smooth fibers and wall action. Furthermore, the density of the mix with PP fibers decreases as compared with the control mortar. Additionally, the increased peak flexural load with SBR latex was due to the improved bond between the cement paste and fine aggregates. The flexural toughness results are in line with the peak flexure loads for ferrocement panels with steel and PVC mesh.

## 5. Conclusions

Flexural strength and flexural toughness were evaluated for ferrocement panels using steel and plastic mesh as reinforcement. Control specimens with PP fibers, specimens with SBR latex, and specimens with a combination of PP fibers and SBR latex were evaluated for both steel and plastic mesh ferrocement panels. The following conclusions are drawn from this research.

- The addition of PP fibers in this study exhibited a reduction in flexural strength of 17.02% for specimen SI and 6.38% for specimen FP. The PP fibers alone had not shown significant contribution in compressive and flexural strength and toughness due to both a weak bond between the fibers and the matrix and less density than the control ferrocement mortar.
- The addition of SBR latex improved flexural strength by 12.59% and 11.01% for iron mesh (SI) and PVC plastic mesh (SP), respectively, which is due to the increased bonding between the cement paste and fine aggregates.
- All the specimens with steel mesh exhibited a discrete center crack, whereas the panels with plastic mesh resulted in smeared cracking and more peak displacement when subjected to the four-point bending test. This shows enhanced energy absorption capacity for the plastic mesh ferrocement panels.

In the present study, PVC mesh was utilized to avoid mesh corrosion and to enhance the service life of panels. However, due to less tensile strength, all ferrocement panels with PVC plastic exhibited a smaller peak load (e.g., 11.3 kN for CI vs. 9.92 kN for CP) as compared to the specimens with welded iron mesh. This decrease in capacity may be addressed via using hybrid meshes, i.e., using PVC mesh in the outer layer and iron mesh in the inner side of the panel, which can be studied in future studies. The concepts used in this study could be extended globally via the use of similar welded iron mesh and comparable PVC mesh and using fine aggregate conforming to ACI 549.1R-18 [47].

## Figures and Tables

**Table 1:** Chemical composition of the cement and fine aggregates.

Material	Constituents (%)						
	LOI *	SiO_2_	SO_3_	CaO	Fe_2_O_3_	Al_2_O_3_	MgO
Cement	2.15	21.45	2.32	62.70	3.75	5.10	1.61
Sand	2.64	88.90	0.05	2.29	1.37	2.85	0.62

* LOI = Loss of ignition.

**Table 2:** Physical properties of sand.

Material	Fineness Modulus ASTM C136 [27]	Bulk Density (kg/m^3^) ASTM C29 [28]	Specific Gravity ASTM C127, ASTM C128 [29,30]	Water Absorption (%) ASTM C127, ASTM C128 [29,30]
Sand	2.21	1590	2.63	1.2

**Table 3:** Properties of reinforcing meshes.

Mesh Type	Average Opening Size	Wire Area	Yield Strength	Ultimate Strength
	(mm)	(mm^2^)	(MPa)	(MPa)
Welded Iron	16.65 × 12.65	2.835	216	272
PVC plastic	14.1 × 14.1	2.71	-	26.3

**Table 4:** Technical detail of PP fiber.

Color	Density	Fiber Length	Fiber Diameter	Melting Point	Water Absorption
	(g/cm^3^)	(mm)	(micron)	(°C)	(%)
Translucent White	0.9	12	15–25	150–170	0

**Table 5:** Chemical and Physical properties of SBR latex (source: vendor).

Appearance	pH	Specific Gravity	Chloride Contents	Viscosity	Particle Size	Freeze/Thaw Resistance	Solid Contents
Emulsion White	9.5–10.5	1.02 ± 0.02 @ 25 °C	Nil	100–500 mpa.s	20 µ	Excellent	47%

**Table 6:** Detail of ferrocement panels.

Specimen Nomenclature	Mesh Type	PP Fiber Dosage (%)	SBR Dosage (%)	Number of Test Specimens
CP	PVC Plastic	-	-	2
FP		0.5	-	2
SP		-	5	2
SFP		0.5	5	2
CI	Welded Iron Mesh	-	-	2
FI		0.5	-	2
SI		-	5	2
SFI		0.5	5	2
Total				16

**Table 7:** Peak loads of ferrocement panels with iron and plastic mesh.

With Iron Mesh					With Plastic Mesh				
Mix	Peak Load (kN)				Mix	Peak Load (kN)			
	Sample-1	Sample-2	Avg.	% Diff.		Sample-1	Sample-2	Avg.	% Diff.
CI	11.3	11.14	11.22	0.00	CP	9.92	10.93	10.43	0.00
FI	9.18	9.44	9.31	−17.02	FP	9.67	9.85	9.76	−6.38
SI	11.69	12.59	12.14	8.20	SP	10.79	11.23	11.01	5.61
SFI	11	11.9	11.45	2.05	SFP	10.94	-	10.94	4.94

**Table 8:** Flexural toughness of ferrocement panels.

With Iron Mesh					With Plastic Mesh				
Mix	Flexural Toughness (kN-mm)				Mix	Flexural Toughness (kN-mm)			
	Sample-1	Sample-2	Avg.	% Diff.		Sample-1	Sample-2	Avg.	% Diff.
CI	264.6	239.2	251.9	0.00	CP	379.1	436.6	407.9	0.00
FI	204.4	237.7	221.1	−12.23	FP	169.8	194	181.9	−55.40
SI	253.9	268.3	261.1	3.65	SP	365.3	361.8	363.6	−10.86
SFI	253.3	253.1	253.2	0.52	SFP	200.9	-	200.9	−50.74

## Data Availability

Data are contained within the article.
